# Transactions of the Medical Society of Virginia

**Published:** 1877-03-20

**Authors:** 


TRANSACTIONS OF THE MEDICAL SOCIETY OF VIRGINIA.—We are in receipt of the *Virginia Medical Monthly*, of the *Transactions of the Medical Society of Virginia*, containing a series of interesting and valuable reports and papers. Dr. Cunningham, of Richmond, the President, made the opening address, which will be found interesting; followed by the address of the Annual Orator, Dr. McDonald, elected for the occasion; his subject "*The Doctor*," is handled in an interesting manner. Then follow able reports of Committees on Advances in Chemistry, Pharmacy, Surgery, Administration of Chloroform, Materia Medica and Therepeutics, Practice of Medicine, Public Hygine, Reports of Cases, constituting the most valuable document of the kind we have had the pleasure of perusing. Our readers would be both interested and profited by procuring and reading a copy of these transactions.

